# Polyphyletic Nature of *Salmonella enterica* Serotype Derby and Lineage-Specific Host-Association Revealed by Genome-Wide Analysis

**DOI:** 10.3389/fmicb.2018.00891

**Published:** 2018-05-17

**Authors:** Yann Sévellec, Marie-Léone Vignaud, Sophie A. Granier, Renaud Lailler, Carole Feurer, Simon Le Hello, Michel-Yves Mistou, Sabrina Cadel-Six

**Affiliations:** ^1^Université PARIS-EST, Agence Nationale de Sécurité Sanitaire de L’Alimentation, de L’Environnement et du Travail (ANSES), Laboratory for Food Safety, Maisons-Alfort, France; ^2^French Institute for Pig and Pork Industry, Le Rheu, France; ^3^Centre National de Référence des Salmonella, Unité des Bactéries Pathogènes Entériques, Institut Pasteur, Paris, France

**Keywords:** *Salmonella* Derby, SNP analysis, AMR analysis, *Salmonella* pathogenicity island, FimH adhesin, host-association’s genetic markers

## Abstract

In France, *Salmonella* Derby is one of the most prevalent serotypes in pork and poultry meat. Since 2006, it has ranked among the 10 most frequent *Salmonella* serotypes isolated in humans. In previous publications, *Salmonella* Derby isolates have been characterized by pulsed field gel electrophoresis (PFGE) and antimicrobial resistance (AMR) profiles revealing the existence of different pulsotypes and AMR phenotypic groups. However, these results suffer from the low discriminatory power of these typing methods. In the present study, we built a collection of 140 strains of *S*. Derby collected in France from 2014 to 2015 representative of the pork and poultry food sectors. The whole collection was characterized using whole genome sequencing (WGS), providing a significant contribution to the knowledge of this underrepresented serotype, with few genomes available in public databases. The genetic diversity of the *S.* Derby strains was analyzed by single-nucleotide polymorphism (SNP). We also investigated AMR by both genome and phenotype, the main *Salmonella* pathogenicity island (SPI) and the *fimH* gene sequences. Our results show that this *S.* Derby collection is spread across four different lineages genetically distant by an average of 15k SNPs. These lineages correspond to four multilocus sequence typing (MLST) types (ST39, ST40, ST71, and ST682), which were found to be associated with specific animal hosts: pork and poultry. While the ST71 and ST682 strains are pansusceptible, ST40 isolates are characterized by the multidrug resistant profile STR-SSS-TET. Considering virulence determinants, only ST39 and ST40 present the SPI-23, which has previously been associated with pork enterocyte invasion. Furthermore, the pork ST682 isolates were found to carry mutations in the *fimH* sequence that could participate in the host tropism of this group. Our phylogenetic analysis demonstrates the polyphyletic nature of the *Salmonella* serotype Derby and provides an opportunity to identify genetic factors associated with host adaptation and markers for the monitoring of these different lineages within the corresponding animal sectors. The recognition of these four lineages is of primary importance for epidemiological surveillance throughout the food production chains and constitutes the first step toward refining monitoring and preventing dispersal of this pathogen.

## Introduction

In the European Union, *Salmonella enterica* subspecies *enterica* serotype Derby (*S.* Derby) is the most abundant serotype isolated from pork. It accounts for 22.9% of all isolates, followed by monophasic strains of *S*. Typhimurium (22.3%) and *S*. Typhimurium (20.6%) (EFSA, 2016). In France, between 2000 and 2015 *S*. Derby ranked between 5th and 8th position (*n* = 164 to 178 clinical isolates) of the most frequently isolated serotypes in humans (Weill and Le Hello, 2014). In the entire food sector, the data of the ANSES *Salmonella* Network (jointly with the National Reference Laboratory) show that this serotype is the 4th most frequently isolated after *S*. Typhimurium, its monophasic variant *S.* 1,4,[5],12:i:- and *S*. Enteritidis (Leclerc et al., 2016). *S*. Derby is principally isolated from pork and poultry meat in France with a prevalence reaching 1.4% for pork and 3.2% for *Gallus gallus* (compared with <1% for turkey) (DGAl, 2014, 2015). All together, these data indicate that *S.* Derby is a significant threat to human health, mainly associated with the pork and poultry sectors.

This serotype is not exclusively adapted to pigs but most often associated with this source (Valdezate et al., 2005; EFSA, 2009). *S.* Derby was recently reported as the most common serotype from turkey flocks in the United Kingdom (∼50% of isolates from pigs and ∼40% from turkeys) (Hayward et al., 2016). In the United Kingdom, between 2014 and 2015 the number of notifications increased more than fivefold in turkey flocks (from 38 to 217 isolates, respectively), showing how well this serotype is adapted to this host (EFSA, 2016).

It is notable that two distinct lineages of *S*. Derby have been identified in the United Kingdom. They differ genotypically and phenotypically by the presence and absence of the *Salmonella* pathogenicity island 23 (SPI-23) and by the higher ability of strains possessing the SPI-23 to invade the porcine jejunum-derived cell line IPEC-J2 (Hayward et al., 2014). These two lineages seem to be adapted to distinct animal sources, probably pig and turkey, but the hypothesis cannot be confirmed because of the limited number of *S*. Derby isolates analyzed (*n* = 16) (Hayward et al., 2016). The presence of different *S.* Derby clonal groups prominent within the food chain was revealed previously in Spain, Germany, and France (Valdezate et al., 2005; Hauser et al., 2011; Kerouanton et al., 2013). These studies suffer, however, from the low discrimination potential of the technique used, pulsed field gel electrophoresis (PFGE) (Valdezate et al., 2005; Kerouanton et al., 2013). A study conducted by the ANSES *Salmonella* Network on a large panel of *S*. Derby strains showed that 42% of the *S*. Derby strains collected since 2005 were assigned to the same profile (SDBYXB0001 for 52/123 strains), highlighting the discrimination limits of PFGE for this serotype with an overall discrimination index of 0.75 (Kerouanton et al., 2007). Contrasting with PFGE studies, recent investigations based on multilocus sequence typing (MLST), clustered regularly interspaced short palindromic repeats (CRISPRs) and whole genome sequencing (WGS) analysis on a small selection of strains suggested that *S.* Derby should be considered as a polyphyletic serotype (Hayward et al., 2016; Zheng et al., 2017). Those studies, however, do not represent the total diversity of the serotype, either because of the limited number of strains analyzed or the low resolution given by the method used.

Considering the prevalence of *S.* Derby in the pork and poultry food sectors, we decided to thoroughly investigate the genetic diversity of this serotype using a WGS approach on a large dataset (*n* = 140) representative of the geographical and source origins in France. In contrast to conventional molecular typing methods, WGS has the potential to compare whole genomes at a single-nucleotide resolution. Methods based on single-nucleotide polymorphisms (SNPs) allow for a detailed, targeted analysis of variations among related bacterial isolates. WGS has recently been postulated to be an ultimate subtyping technique (Gilchrist et al., 2015; Dunn, 2016), and SNP-based cluster analysis was already successfully used to explore the genomic diversity of *Salmonella* isolates across serotypes as well as among and within specific food sources (Wilson et al., 2016; Ferrari et al., 2017).

The strains of this collection were isolated in 2014 and 2015 from the pork and poultry sectors, which are the main sources of this serotype, to obtain a comprehensive view of their distribution. The collection was investigated by MLST and SNP analysis. Because resistance of *Salmonella* to antimicrobial agents is a worldwide problem, and antimicrobial resistance (AMR) has already been described in *S*. Derby isolates (Valdezate et al., 2005; Hauser et al., 2011; Keelara et al., 2013; Kerouanton et al., 2013), susceptibility tests were performed and identification of acquired AMR genes was also investigated. The detection and characterization of *Salmonella* pathogenicity islands (SPI-1 to 5 and SPI-23), coding for virulence factors implied in adhesion and invasion of the host, and of the *fimH* gene, known to be a marker of the host specificity within *Salmonella*, were also investigated to identify potential genome signatures responsible for host specificity of the Derby serotype for pig and poultry.

## Materials and Methods

### Strain Selection

A panel of 140 *S*. Derby strains was selected from all the isolates received by the *Salmonella* Network in 2014 and 2015 (*n* = 598) (Leclerc et al., 2016). The epidemiological data of the 140 *S*. Derby strains are listed in **Supplementary Table S1**. Duplicates (isolates with the same sampling date, geo-localization, and isolation matrix) and isolates from animals and feed were excluded. The strains selected came principally from the food sector (from slaughterhouses to the retail market). Only pork and poultry meat categories were considered, because of the low prevalence of *S*. Derby (<1%) in beef and cattle (Leclerc et al., 2016). Within the 140 strains, the proportion of strains from pork and poultry meat (85 and 60, respectively) was chosen in line with French production data. The production of pork and poultry meat accounts for 38.3 and 29.4% of French meat production, respectively (Menard et al., 2015). For each sector, the number of strains was selected, in each region, proportionally to its production compared to the total French national production of meat products (**Supplementary Table S1** and **Supplementary Figure S1**). For poultry, since the *S*. Derby strains were concentrated in Brittany, all strains belonging to other regions were incorporated into the collection.

All strains were identified as belonging to the Derby serotype by glass slide agglutination, according to the White-Kauffmann-Le Minor scheme (Grimont and Weill, 2007).

### Genomic DNA Preparation and Sequencing

DNA was prepared from 10 ml of BHI overnight cultures with the Wizard^®^ Genomic DNA Purification Kit (Promega, France) according to the manufacturer’s instructions for gram-negative organisms. Gels of 1.5% agarose were used to assess the quality of the extraction (and an eventual degradation of the DNA). The DNA concentration was measured with a Qbit^®^ fluorimeter and the purity ratio was assessed with a Nanodrop^®^ Spectrophotometer. Libraries were prepared and the NGS sequencing were performed by the *Institut du Cerveau et de la Moelle épinière* (ICM)^1^ (Hôpital de la Pitié-Salpêtrière, Paris). Each individual library (batch of 96 DNA) was prepared with the Nextera XT technology (Illumina). The indexing of the DNA was carried out during the construction of the library. The libraries were purified with the Agencourt AMPure XP system (Beckman Coulter) and quantification was performed using the Microfluidic LabChip GX (PerkinElmer).

The sequencing of the final library (DNA 96) by NextSeq 500 was carried out using a 300 cycle High Output kit v2 cartridge (400 million paired reads and 800 million single reads in 150 bases). Each Illumina paired-end sequence contained 300 base pairs (bp) (reads are 150 bases). The minimum theoretical coverage is of 30×–50×.

### Multilocus Sequence Typing (MLST)

The seven housekeeping gene sequences (*aroC, dnaN, hemD, hisD, purE, sucA*, and *thrA*) for each strain were detected using the MLST service of the Center for Genomic Epidemiology (CGE)^2^, which enabled us to determine the sequence type (ST) directly from the read files.

### Single Nucleotide Polymorphism (SNP) Analysis

The SNP analysis was conducted using the VARCall workflow (Felten et al., 2017). In the absence of a complete reference *S.* Derby genome sequence and since VARCall requires a closed reference genome, sequence reads were mapped to *Salmonella* Typhimurium LT2 (NCBI NC_003197.1). The VCF files computed by VARCall were combined into a merged VCF which was filtered using Samtools and Picard tools to eliminate duplicated regions and variants solely linked to the reference genome. A pseudogenome obtained on the reference was generated using the Genome Analysis ToolKit (GATK) (McKenna et al., 2010), SNP and inDEL were predicted, and the distance matrix between each pair of genomes was calculated.

Phylogenetic analyses on the dataset were computed using RaXml software (Stamatakis, 2014). The phylogenetic trees were constructed under the maximum likelihood criterion using the GTR-gamma model of nucleotide evolution. The phylogenetic analyses were based on the pseudogenome obtained using the GATK. The phylogenetic data were visualized using interactive Tree Of Life (iTOL^3^) (Letunic and Bork, 2016).

### Statistical Analyses

The non-normality of the data (number of paired SNP differences) was assessed using the Shapiro test (Royston, 1995) on R from the pairwise matrix generated by the VARCall workflow described above. The comparison between the paired SNP differences was tested by a Kolmogorov–Smirnov test (KS-test) (Huang et al., 2016), to find the variance of the distribution by paired SNP differences, which had been proven significantly unequal by the Fisher test (Markowski and Markowski, 1990).

### Identification of Acquired Resistance Genes

The whole panel of genomes was analyzed using the ResFinder 2.1 application (Zankari et al., 2012) on the CGE server. The threshold for reporting a match between a gene in the ResFinder database and the input *S*. Derby genome was set at 90% identity over at least 3/5 of the length of the resistance gene. BioNumerics software version 7.6.1 (Applied Maths, Sint-Martens-Latem, Belgium) was used to perform a BLAST to localize each resistance gene inside the assembled genome. In order to investigate the implantation of the AMR gene within the genome of *S.* Derby, SGI-1 coding sequences (NCBI:AF261825.2) were extracted and BLASTed against the dataset with the BioNumerics BLAST tool. The complete genomic sequence of the SGI-1 was investigated using the BioNumerics alignment and sequence visualization tools.

### Antimicrobial Susceptibility Tests

Strains were selected by their genetic distance: for the pork sector, all strains above a cut-off defined as the median genetic distance (110 SNPs) were selected (*n* = 40). The same cut-off (36 SNPs) was set to select ST71 strains from the poultry sector (*n* = 21).

Antibiotic susceptibility was determined using the disk diffusion method as recommended by the Clinical and Laboratory Standards Institute (CLSI) (CLSI, 2015, 2016). Fifteen antimicrobials (Bio-Rad, Marne-la-Coquette, France) were tested: amoxicillin/clavulanic acid (AMC; 30 μg), ampicillin (AMP; 10 μg), cephalothin (CEF; 30 μg), cefotaxime (CTX; 30 μg), ceftazidime (CAZ; 30 μg), chloramphenicol (CHL, 30 μg), sulfonamides (SSS; 300 μg), trimethoprim-sulfamethoxazole (SXT; 1.25+23.75 μg), streptomycin (STR; 10 U), gentamicin (GEN; 10 μg), kanamycin (KAN; 30 UI), tetracycline (TET; 30 UI), nalidixic acid (NAL; 30 μg), ciprofloxacin (CIP; 5 μg), pefloxacin (PEF; 5 μg). A Colistin disk (CST; 10 μg) was used on each plate for quality management purposes to ensure the absence of contamination. Automatic readings were performed using the BIOMIC^®^ V3 system (Giles Scientific Inc., Santa Barbara, CA, United States). Isolates were classified as susceptible, intermediate, or resistant according to the clinical interpretive criteria recommended by CLSI (2016).

### Salmonella Pathogenicity Islands (SPI) – Identification

SPI-1, the two segments of SPI-2, SPI-3, SPI-4, SPI-5, and SPI-23 were tested for within the set of 140 genomes of *S*. Derby using BLASTn^4^ with a cut-off of 90% identity. The SPI reference sequences used for the BLASTs were collected by the NCBI database (accession numbers KP279311.1, AJ224978.1, KP258194.1, AF106566.1, KP234070.1, AY144492.1, and LAZB00000000-project PRJNA270707, respectively). All these sequences correspond to complete SPIs from *S.* Typhimurium strains with the exception of SPI-23, which corresponds to *S*. Derby strain 07CR553 (Kerouanton et al., 2015).

In the absence of a reference sequence for SPI-23, the primers defined by Hayward et al. (2016) were used to generate the *in silico* PCR and to extract a complete SPI-23 sequence from the genome of the *S.* Derby strain 07CR553 (accession number LAZB00000000), contig 5 (1894692..1931302) (Kerouanton et al., 2015). Both the complete sequence of SPI-23 obtained as described above and the 41 Coding DNA Sequences (CDS) described by Hayward were BLASTed separately against our dataset to verify the results of the *in silico* PCR.

### Fimbriae FimH Allele Characterization

The fimbriae FimH allele, identified as host specificity marker by previous studies (Yue et al., 2015), was also sought and characterized within our panel of *S*. Derby genomes. The *fimH* gene sequence was isolated from the project PRJNA297164 (NCBI) and compared with the sequence of the dataset using BLASTn.

As an element of comparison, 25 *fimH* sequences from 25 different serotypes of *Salmonella enterica* subsp. *enterica* selected among the most frequently isolated in humans, animal, and food (Weill and Le Hello, 2014; EFSA, 2016) were added to this study (**Supplementary Table S2**). Whole FimH alleles were extracted by dataset, annotated using the BioNumerics 7.6.1 annotation plugin (Applied Maths, Sint-Martens-Latem, Belgium) and aligned on the reference *fimH* sequence of the *S.* Typhimurium strain SL1344 (accession NC_016810.1). The alignment was carried out using the BioNumerics 7.6.1 alignment tool and the mutations were identified using the mutation prediction tool of the same software.

## Results

### MLST Profiles

**FIGURE 1:**
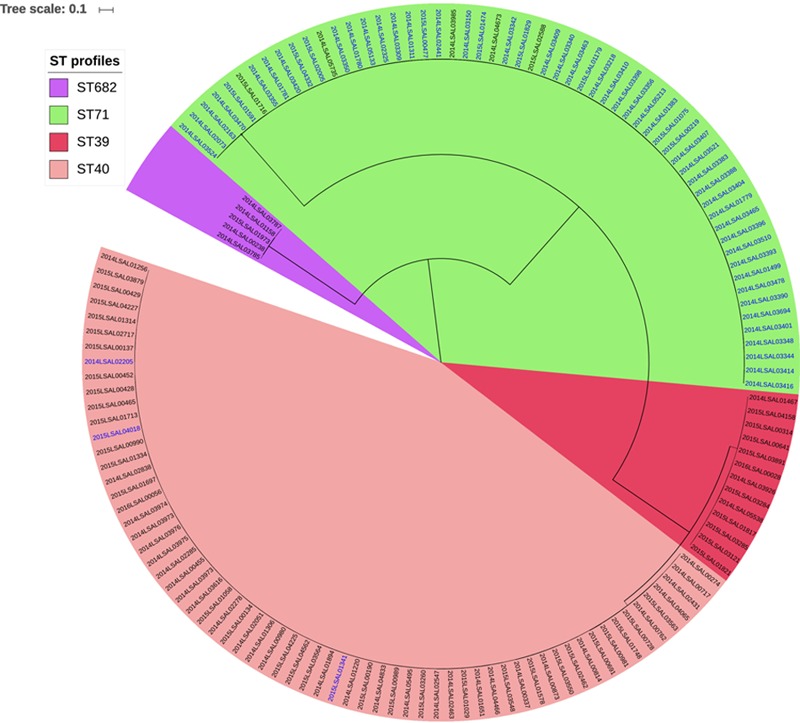
Phylogenetic SNP tree for 140 strains of *S.* Derby constructed under the maximum likelihood criterion using the GTR-gamma model. The scale bar indicates the number of substitutions per site. Four clusters were identified and named ST39, ST40, ST71, and ST682 with reference to the MLST profiles with which the correspondence is perfect. The labels in blue correspond to the strains of the poultry sector. The black labels correspond to the strains of the pork sector.

Four different MLST profiles, ST40, ST39, ST71, and ST682, were identified among the 140 studied genomes. ST40 and ST39 were distinct by only eight mutations in the *sucA* locus and could therefore be included in the same eBURST complex (Achtman et al., 2012). The most frequent profile within the collection was ST40 (*n* = 64 genomes) followed by ST71 (*n* = 58), ST39 (*n* = 13), and ST682 (*n* = 5) (**Figure 1**). ST40 grouped together 61 strains isolated from pork meat (95.3%) and 3 from poultry (4.7%). The ST39 and ST682 strains were isolated from pork (13 and 5 strains, respectively). ST71 was represented by 54 strains isolated from poultry meat (92%) and 4 strains from pork meat (8%).

### Phylogenetic Analysis of *S.* Derby Strains Panel

Four clusters were obtained by the SNP analysis of the 140 genomes of *S.* Derby analyzed (**Figure 1**). As shown in **Figure 1**, the SNP analysis clustered the 140 genomes in four groups. These four groups were fully consistent with the ones identified by MLST analysis (100% identity). To simplify the comprehension of the results, these 4 lineages were named with their ST profiles (ST39, ST40, ST71, and ST682).

**FIGURE 2:**
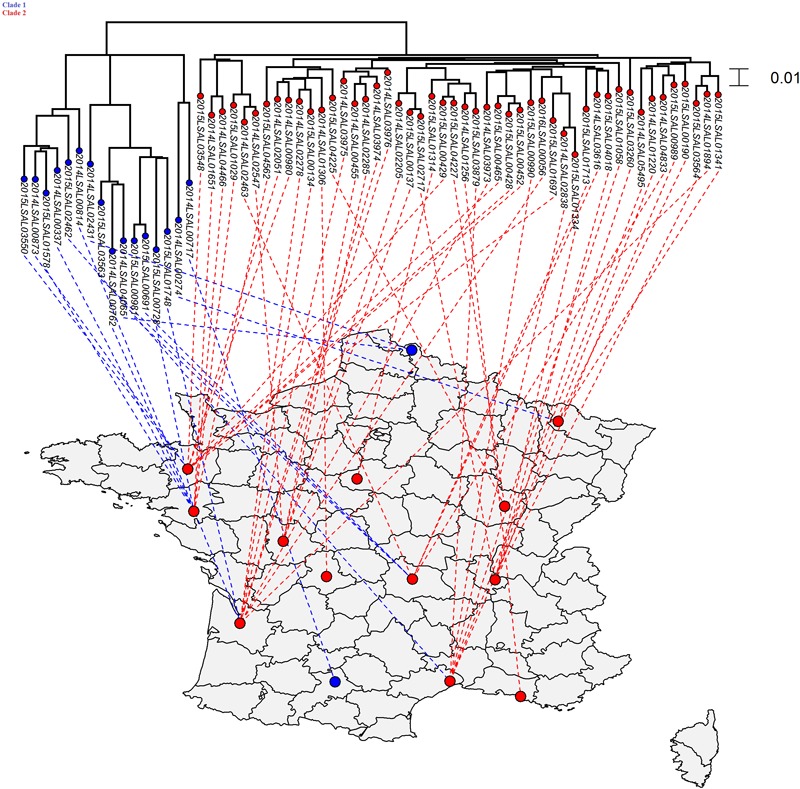
Phylogenetic SNP distribution of the strains from the cluster ST40 in relation with their geographical repartition. Tree was constructed under the maximum likelihood criterion using the GTR-gamma model. The scale bar indicates number of substitutions per site. Two distinct clades were identified. There is no apparent correlation between geographical and phylogenetic distribution of the different strains belonging to these two clades.

**FIGURE 3:**
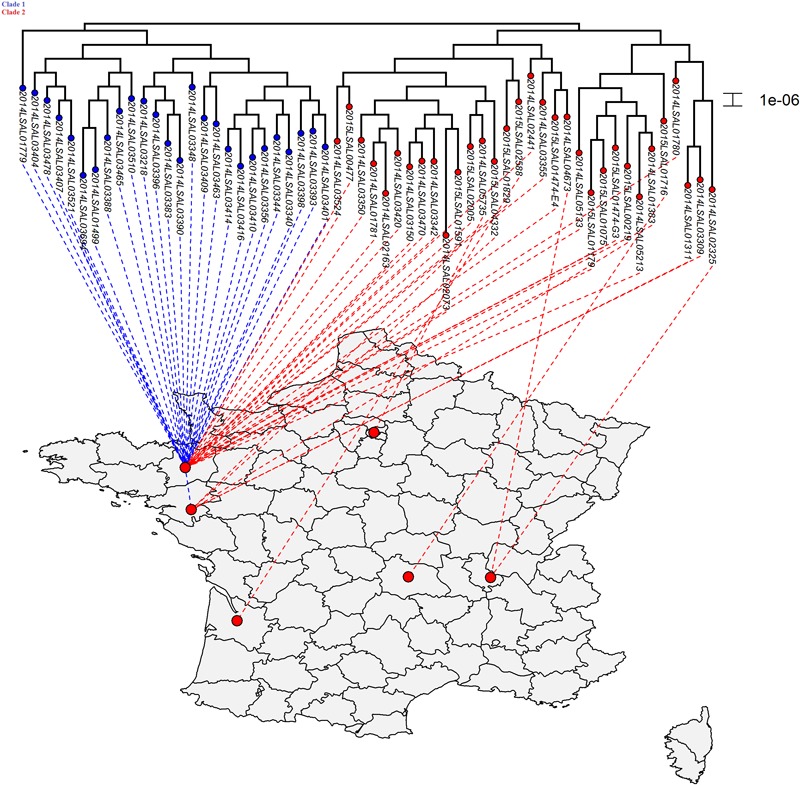
Phylogenetic SNP distribution of the strains from the cluster ST71 in relation with their geographical repartition. Tree is constructed under the maximum likelihood criterion using the GTR-gamma model. The scale bar indicates the number of substitutions per site. Two distinct clades were identified. The strains belonging to Clade 1 were principally isolated in Brittany (25/26 strains).

Within each of these groups, genomes were found to differ by less than 300 SNPs. The genomes belonging to ST39 were most closely related to ST40 genomes with an average of 3,962 SNPs and a standard deviation (SD) of 20 SNPs. The strains belonging to the ST71 cluster were distant from the ones belonging to ST40 by 26,957 SNPs, with an SD of 1,583. The ST profile 682 was the most genetically distant from ST39, ST40, and ST71 with an average of 33,961 SNPs and an SD of 4,102. Considering epidemiological information, there was no evidence of a relationship between the genomic proximity and geographical localization for the different lineages. Closely related strains could have very different geographical origins and vice versa. It is also the case for the ST39 and ST682 lineages, which grouped together only 13 and 5 genomes, respectively. These lineages presented a very wide geographic distribution, clustering together strains from Pays-de-la-Loire, Bourgogne, Languedoc-Roussillon and Centre. The Pays-de-la-Loire region, however, presented considerable diversity in terms of ST profiles with strains from the four different lineages (**Figures 2, 3** and **Supplementary Figure S2**). Consistent with the distribution of the pork and poultry food production chains in France, ST40 was geographically more widespread than ST71. Our results clearly associated three lineages with the pork sector (ST39, ST40, and ST682) and one with the poultry sector (ST71).

### Phylogenetic Analysis of the ST40 Cluster

As shown in **Figure 2**, the strains forming the ST40 cluster could be divided into two clades. Clade 1 contained 16 strains with an average of 192 SNPs and an SD of 83 SNPs while Clade 2 contained 48 strains with an average of 68 SNPs and an SD of 16. These two clades were separated by an average of 135 SNPs and an SD of 64. A Kolmogorov–Smirnov test between these two groups statistically confirmed the distinction between those two clades (*p*-value ≤ 2.2.10^-16^). There is no apparent correlation between geographical and phylogenetic distribution of the different strains belonging to these two clades.

### SNP Inference for the ST71 Cluster

The ST71 cluster contained genomes presenting only an average of 28 SNPs and an SD of 10 (**Figure 3**). The statistical analysis showed that two clades could be identified (*p*-value = 6.10^-13^) in the ST71 cluster. Between two genomes, in Clade 1 there was an average of 19 SNPs (SD of 5) while in Clade 2 there was an average of 34 SNPs (SD of 14). There was no apparent chronological relationship between the different strains belonging to these two clades. Considering the geographical distribution of the sampling (**Supplementary Figure S2**), the strains belonging to Clade 1 were principally isolated in Brittany (25/26 strains).

### Antimicrobial Susceptibility Tests and Detection of Acquired Resistance Genes

**FIGURE 4:**
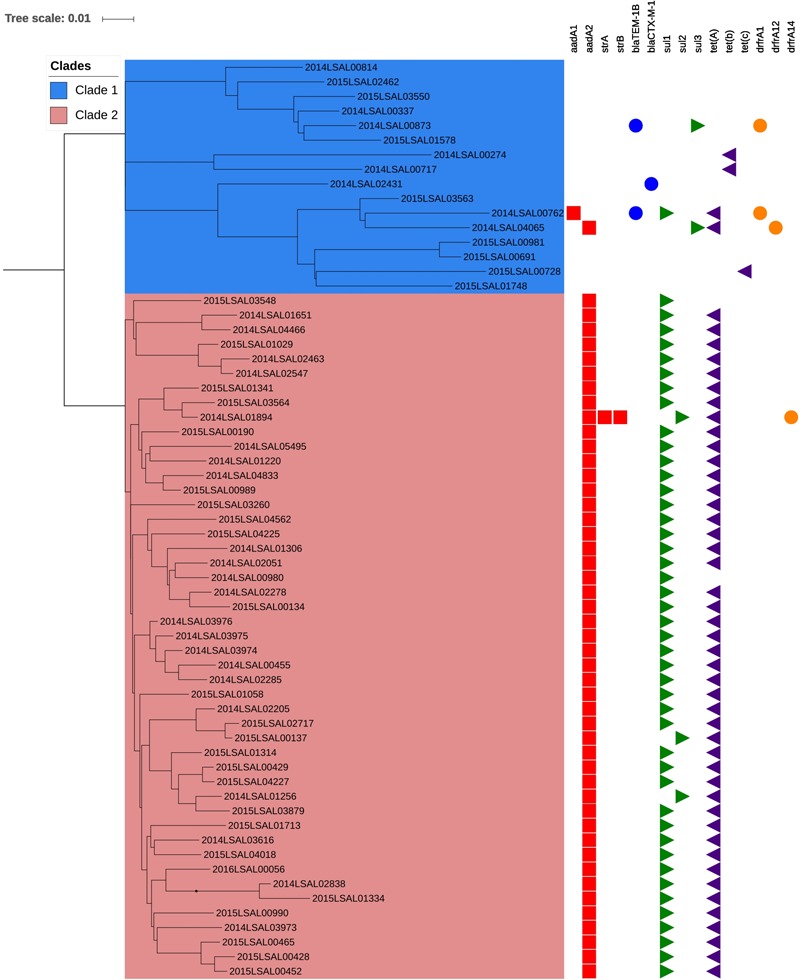
Phylogenetic SNP tree of the strain from the cluster ST40. The scale bar indicates the number of substitutions per site. The box in blue corresponds to Clade 1 and the box in red to Clade 2. The predicted antibiotic resistance genes are indicated on the right. The red cubes correspond to the resistance gene to aminoglycosides antibiotic, the blue circle to beta-lactam, the green triangle to sulfonamides, the purple triangle to tetracycline, and the orange circle to trimethoprim.

Complete and partial antibiotic resistance genes identified in the 140 *S*. Derby genomes studied are listed in **Supplementary Table S3**. No resistance gene was detected in 59% (83/140) of the studied genomes. Among the 57 genomes harboring AMR genes, 81% were simultaneously carrying *aadA2, sul1*, and *tetA* (**Figure 4** and **Supplementary Table S3**). These genes mediate resistance to aminoglycosides, sulfonamides, and tetracyclines, respectively. This expected phenotype had been confirmed phenotypically for 46 “STR-SSS-TET” strains belonging to Clade 2 of ST40 (**Figure 4**). The resistance genes carried by the strains of Clade 1 of ST40 were more diverse. Most of the ST71, ST39, and ST682 strains were found to be carrying no AMR genes, 97% (57/58), 100% (13/13), and 80% (4/5), respectively.

### Distribution and Diversity of SPI-1, -2, -3, -4, -5

**Table 1** compiles the information about the length, number of genes, and percentage of GC content in the different SPIs. SPIs 1 to 5 are present in the 140 genomes studied. The alleles are identical within each lineage (100% of identity) but can be different between lineages. ST40 and ST39 share the same alleles (100% identity) with the exception of 6 genes in SPI-5 (*pipB, pipC, pipD, sopB, copS*, and *copR*). The global conservation of SPIs 1 to 5 is high between the four Derby lineages, with an overall sequence identity above 97% and allelic difference that does not exceed four substitutions. The genes *sseB, sseC*, and *sseD* of the second region of SPI-2 present the highest allelic difference between the lineages, with a sequence identity between 94.91 and 97.80%. The gene *rhuM* at the left-hand end of SPI-3 is only present in ST682. This gene (coding for a hypothetical protein) and *sugR* (coding for a putative ATP binding protein) are absent in ST40 and ST39 and only present as a fragment in ST71 (corresponding to 1..1246 out of a 1560 bp long gene) and ST682 (one fragment of 520 bases corresponding to the beginning of the gene *sugR* and a second fragment of 535 corresponding to the end of the gene *sugR*). An integron previously described by Amavisit et al. (2003), containing seven genes related to the adhesion structures, pili and fimbriae (*ecpD2, ecpD1, htrE, yadM, yadL, yadK*, and *yadC*), flanked by two truncated transposase genes (*tpase2* and *tpase1*), was detected in the SPI3 of ST71, ST40, and ST39.

**Table 1:** Length, genes, and GC content of the main SPIs for *Salmonella*.

*Salmonella* pathogenicity island	Length (Kb)	GC%	Total number of genes	ST40 and ST39	ST71	ST682	Range of identities (%) between the four lineages
SPI-1	38	45.77	39	39/39	39/39	39/39	98.01 to 100
SPI-2 region 1	12	53.05	10	10/10	10/10	10/10	98.10 to 100
SPI-2 region 2	25	40.00	31	31/31	31/31	31/31	94.91 to 100
SPI-3	16	51.42	10	8/10^a^	9/10^b^	9/10^c^	95.43 to 100
SPI-4	27	45.18	10	10/10	10/10	10/10	97.70 to 100
SPI-5	9	45.50	8	8/8	8/8	8/8	97.27 to 100
SPI-23	36	37.91	41	41/41	0/41^d^	0/41	99.90 to 100

*^*a*^Missing *sugR* and *rhuM*; ^*b*^Truncated *sugR* and missing *rhuM*; ^*c*^Missing *sugR* (fragment only); ^*d*^ Except the strain 2014LSAL05133 which present fragments of SPI-23.*

### Distribution and Diversity of the SPI-23 Region

SPI-23 was only present in the sequences of *S*. Derby belonging to ST40 and ST39 with a sequence identity of 100% or above to 99.9% (for a 100% coverage). This *Salmonella* pathogenicity island was missing in ST682 strains and in most ST71 strains with the exception of strain 2014LSAL05133, which only contains fragments of SPI-23. The *docB* gene, reported to end SPI-23, was present in all strains. We do not believe that the assembly process could alter the reconstruction of SPI-23, because all ST39 and ST40 strains were found to carry the 41 CDS (with 100% of identity) constitutive of SPI-23 and used as references for BLAST analysis. The results obtained by *in silico* PCR with the primers described by Hayward et al. (2016) are shown in **Supplementary Figure S2**.

### FimH Gene Alleles

**FIGURE 5:**
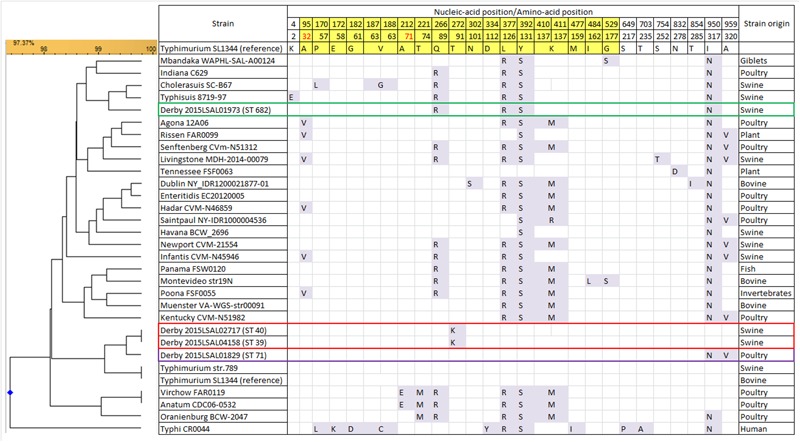
Variant residue positions in the FimH protein sequence for 25 serotypes and some *S*. Derby strains of this study. The results show a 100% identity of the *fimH* sequences inside each ST lineage. Only three strains of each group are represented in the comparison, as examples. The residues belonging to the lectin domain (23–196, yellow background) and the pilin domain (199–335, white background) are framed, for a total of 27 missense SNPs (23 already described and 4 new ones) for a total of 17 mutations of the lectin domain and 9 of the pilin domain. The residues in red are known to be part of the mannose binding pocket.

FimH amino acidic sequences of the four *S*. Derby lineages obtained in the present study were compared with those of strains previously isolated from swine, poultry, and cattle and belonging to the most frequent serotypes isolated in Europe (Weill and Le Hello, 2014; EFSA, 2016). The results show that there are three different *fimH* alleles in our collection, grouping together ST39 and ST40 isolates, while ST71 and ST682 display a peculiar *fimH* allele-type. FimH protein is characterized by two domains, the lectin and pilin domains. The sequence of the lectin domain of the strains belonging to the poultry-associated ST71 shows 100% identity with that of the *Salmonella* Typhimurium reference strain SL1344. The sequences of strains belonging to ST39–ST40 and ST682 differ by one and two substitutions, respectively, from the SL1344 sequence. The sequence of the pilin domain of T39-ST40 isolates differs by one substitution at position 91 from the SL1344 sequence. The ST71 FimH sequence differs by two missense substitutions from ST39–ST40 and three substitutions from ST682 (**Figure 5**). Considering FimH sequence variations among the 25 most prevalent serotypes, *S*. Derby ST39–ST40 and ST71 are closely related to *S.* Typhimurium, while ST682 is closely related to *S*. Choleraesuis and *S*. Typhisuis with (4/6 and 4/5, respectively) missense mutations and (15/19) silent mutations in common. The residues predicted to be part of the mannose-binding pocket (positions 32 and 71) are conserved in the genomes of all *S*. Derby lineages. The unique substitution found in ST40 and ST39 and not encountered in the sequences of the other serotypes is located at position 272 and is predicted to change amino acid residue 91 from Threonine to Isoleucine. The ST71 has two missense mutations in position 950 (changing residue 317 from Threonine to Asparagine) and 959 (changing residue 320 from Alanine to Valine). Otherwise, the three ST groups ST40, ST39, and ST71 share 6 identical silent mutations. The ST682 clade is more distant from the *fimH* sequence of *S*. Typhimurium SL1344 and displays 16 silent mutations and 4 missense mutations at positions 266 (changing residue 89 from Quinine to Arginine), 377 (changing residue 126 from Leucine to Arginine), 392 (changing residue 131 from Tyrosine to Serine), and 950 (changing residue 317 from Threonine to Asparagine).

## Discussion

### Genetic Diversity of *S.* Derby

The collection of *Salmonella* Derby strains used in this study was chosen to be representative of the diversity in the French food sector. The strains were essentially collected in the west/south-west parts of France corresponding to the most intensive pork and poultry meat production regions.

The phylogenetic analysis reveals that four major *Salmonella* Derby genetic lineages cohabit in France, corresponding to the MLST profiles ST40, ST39, ST682, and ST71. Two of these lineages, ST39 and ST40, differ by 3,962 SNPs (SD of 20 SNPs). This genetic proximity is confirmed by considering their MLST alleles, which can be grouped into the same MLST eBURST complex. The other lineages are much more distant from each other and display an overall inter-group difference of 14,866 SNPs with an SD of 12,961. ST682 is the most genetically different, with an average distance of 33,961 SNPs (SD of 4,102) from the three other groups.

These results highlight that strains displaying the same antigenic pattern *S*. 1,4,[5],12: f,g: [1,2] according to the White-Kauffmann-Le Minor scheme (Grimont and Weill, 2007) and consequently grouped under the same *Salmonella* Derby serotype include at least four genomic lineages. Like studies on *Salmonella* Newport (Timme et al., 2013), our results obtained on a large number of strains provide another example of a polyphyletic serotype.

The four MLST profiles, ST39, ST40, ST71, and ST682, have already been described (Hauser et al., 2011; Wang et al., 2011; Cai et al., 2016; Hayward et al., 2016; Zheng et al., 2017). Hauser et al. (2011) reported ST682 strains in Germany that were involved in a 2013–2014 outbreak associated with the consumption of pork (Simon et al., 2017). Isolation of ST40 strains has been previously described in England, Germany, Midwestern United States, and China, making this ST the most frequently reported in the literature (Hauser et al., 2011; Wang et al., 2011; Cai et al., 2016; Hayward et al., 2016; Zheng et al., 2017). Recently, Zheng et al. (2017) described a collection of 92 strains isolated from the swine and pork production chain in China, almost one third of which belong to ST71. This result contrasts with ours where all ST71 strains were associated with the poultry sector and none was isolated from the pork sector. However, Chinese isolates came from slaughterhouses and retail markets and never from the swine and farm environment, leading to the hypothesis that cross-contamination may have occurred on sites. Otherwise the ST71 was described by Hayward et al. (2016) and associated with turkey meat.

Our work is the first large survey at the serotype level taking into account the different sectors with which *Salmonella* Derby has been associated and that clearly demonstrate the polyphyletic nature of the Derby serotype by whole genome analysis. It further demonstrates through a large national sampling the strong host-association of the four identified lineages. Within our panel the lineages ST40, ST39, and ST682 are associated with pork while ST71 is associated with poultry. Our results were obtained on a large national collection presenting a wide diversity of sampling origins. It can, however, be considered limited due to the time scale (2 years) and the restricted national area and, of course, it does not preclude that additional, uncharacterized lineages could express the Derby antigens. However, our collection brings together in a coherent way, all the genetic diversity that was previously described in a series of separate studies (Hauser et al., 2011; Wang et al., 2011; Cai et al., 2016; Hayward et al., 2016; Zheng et al., 2017).

The phylogenetic analysis carried out on ST40 and ST71, regrouping 87% of the strains analyzed, allowed us to further discriminate four, and statistically supported, clades. The ST40 can be divided into two clades. Clade 1 is highly heterogeneous regarding the antimicrobial profile; it contains susceptible strains and a diversity of antimicrobial-resistance profiles, while Clade 2 is characterized by strains showing a highly homogeneous AMR pattern, STR-SSS-TET (streptomycin, sulfonamides, and tetracycline). The strains clustered in Clade 2 are genetically less distant than the strains of Clade 1, with an average of 68 (SD of 16) and 192 (SD of 83) SNPs, respectively. ST71 can also be divided into two statistically different clades with no differences concerning AMR. The strains belonging to these two clades are essentially pansusceptible.

The ST40 lineage is found throughout France and strains from Clades 1 and 2 were isolated in various links of the food chain: slaughterhouses, secondary processors, retail sale, and food. In light of our results, it would be interesting to acquire information on the genetic diversity of *S.* Derby at the farm level, which is currently lacking. There is a widely held hypothesis that pork becomes contaminated at slaughterhouses supported by several publications (Rostagno et al., 2003; Botteldoorn et al., 2004; Valdezate et al., 2005; Buncic and Sofos, 2012). A recent study by Fois et al. (2017), on the occurrence and the characterization of *Salmonella* strains in slaughtered pigs in Italy, shows self-contamination for 71.5% of *Salmonella*-positive carcasses. Contaminated tools used for slaughtering can participate in the dissemination of this pathogen between carcasses. Contamination can also occur by the use of contaminated tools and work surfaces in the meat-cutting workshops. Above all, an understanding of the modes of transmission within the food production chains and the monitoring of the different lineages throughout the pork and poultry sectors would help national and international health, food, and agricultural authorities to establish suitable hygiene practices against the spread of this pathogen.

The main geographical origin of ST71 is from Brittany and Pays-de-la-Loire, where 76% of the poultry industry in France and 80% of the slaughterhouses are concentrated. Our phylogenetic analysis does not differentiate Derby strains isolated from turkey from those isolated from *Gallus gallus*. The similar prevalence of Derby in the two animal species, 12.6% for *Gallus gallus* and 14.2% for turkey (DGAl, 2014, 2015), suggests that the ST71 strains are well adapted to both *Gallus gallus* and turkey. On the other hand, these data underline the disproportion observed in France between the prevalence of Derby upstream of the food chain (in animals) and the contamination recorded downstream (in food). In our panel representative of the food production chain in France, two strains were isolated from the turkey food sectors and 54 from the *Gallus gallus* food sector. This discrepancy could be explained by the fact that 81% of the production in the French poultry sector concerns *Gallus gallus* and only 5% turkey, and that the farm environment is often common for the two species, so that cross contamination should not be excluded.

### Antimicrobial Susceptibility

Antimicrobial resistance is strongly associated with lineage ST40 in this study, as only 14% of the strains belonging to this lineage are pansusceptible. Most of ST71, ST39, and ST682 strains have no known resistance gene. Clade 2 of ST40 includes the majority of the strains presenting the STR-SSS-TET profile (*n* = 46/48). The STR-SSS-TET profile was first described in Spain (Valdezate et al., 2005). It has also been described in France by Kerouanton et al. (2013) for strains isolated from pig, pork, and humans (accounting for 66.7% of the isolates collected in 2006–2007). Aminoglycosides, sulfonamides, and tetracyclines are the most used antimicrobial classes in the pig sector in France (Binh et al., 2009; Méheust et al., 2016). The exploration of the genome of the ST40 Clade 2 strains revealed the presence between the *trmE* and *yidY* genes of a *Salmonella* genomic island (SGI-1) specific to this clade. This SGI-1 contained a class1 integron delimited by *intI1* and IS1326 insertion sequences. This integron is similar to the class1 integron containing the *aadA2* and *sul1* genes of the SGI-1C described by Beutlich et al. (2011). The SGI-1 also included the *tetA* gene and a cluster of mercuric resistance genes (*merA, merC, merP, merT*, and *merR*) located in a Tn7 transposon.

Interestingly, several studies have shown that the soil plays an important role in the dissemination within microorganisms of IncN, IncW, IncP-1, and pHHV216-like plasmids carrying resistance genes (Binh et al., 2008). The practice of field application of piggery manure, which harbors a substantial reservoir of broad-host-range plasmids conferring multiple antibiotic resistance genes, has been demonstrated to be responsible for this dissemination into agricultural soils, favoring horizontal gene transfer (Binh et al., 2009). Clinically relevant Class 1 integrons are also introduced into soil via similar practices (Aminov, 2011; Colavecchio et al., 2017; Pornsukarom and Thakur, 2017).

Finally, 98% (56/57) of the experimental AMR phenotypes were in agreement with the predictions made by ResFinder. One strain (2014LSAL04065) contains a gene coding for trimethoprim resistance (*dfrA12*), as trimethoprim has only been tested in combination with sulphonamides (SXT), the expression of this trimethoprim resistance phenotype hasn’t been validated. Our results highlight the complementarity of these two analyses, the conjunction of WGS and phenotypic approaches could indeed be able to detect new AMR mechanisms. These affirmations are concordant with the conclusions exposed in the EUCAST review for the antimicrobial susceptibility of *Salmonella* (Ellington et al., 2017). A WGS approach can provide rapid identification for well-known and characterized AMR mechanisms. However, no WGS approach is so far able to predict antimicrobial susceptibility.

### Virulence Factors and Host Specificity

The results concerning the *Salmonella* pathogenicity island -1, -2, -3, -4, -5 showed that all these genomic regions were detected within the panel of *S*. Derby strains analyzed, with an average sequence identity of 97%. The observed sequence differences between the lineages for the genes *sseB, sseC*, and *sseD* coding for the SPI-2 translocon that influences the capacity of type III secretion system (T3SS) protein to invade host cell cytoplasm, could impact the ability of the four *S.* Derby lineages to efficiently multiply in host cells and to survive in macrophages (Wilson et al., 2007; Reynolds et al., 2014). Even if the substitutions were conserved inside each lineage and could be used to discriminate between the different lineages of *S.* Derby, only cellular test experiments could confirm this hypothesis. The SPI-3 was the most variable SPI within the genomes analyzed. We observed a deletion of the left-terminal genes *rhuM* and *sugR* that had been previously reported in several studies as a common deletion in several serotypes such as Derby, Infantis, Virchow, Havana, Newport, and Albany (Beutlich et al., 2011; Figueiredo et al., 2015; McWhorter and Chousalkar, 2015). The *rhuM* gene was present only in the lineage ST682. It is known that *rhuM* gene deletion causes a significant decrease of the epithelial cell invasion capacity (Tenor et al., 2004). The *sugR* gene was found partial or fragmented in all the lineages. ST40, ST39, and ST71 possessed an integron in their SPI-3 containing seven genes related to the adhesion structures, pili and fimbriae genes that were reported as characteristic of *S.* Derby (Amavisit et al., 2003). These results highlight the genomic difference between ST682 and the other *S.* Derby lineages, but as a whole it could be concluded that the genetic distinction between the four Derby lineages does not reside in these pathogenicity islands.

The *Salmonella* pathogenicity island 23 (SPI-23) described by Hayward et al. (2013) has been shown to play a role in adhesion and invasion of porcine tissues (Hayward et al., 2014). The presence of this particular SPI helps explain the host pig specificity of the strains belonging to the lineages ST39 and ST40. The SPI-23 is absent in the strains belonging to the lineage ST71 associated with poultry and in the lineage ST682 related to pig. It has been shown that in the United Kingdom two distinct lineages of *S*. Derby coexist and these two lineages seem to be adapted to distinct sources (pig and turkey), being distinguished by the presence and absence of SPI-23 and the ability to more efficiently invade the porcine jejunum derived cell line IPEC-J2 (Hayward et al., 2016). The results of this study support the hypothesis that the differences in host ranges of *S.* Derby are adaptations to pathogenesis, environmental persistence, and the use of metabolites abundant in their respective host environments.

FimH adhesin, located on type 1 fimbriae, mediates the adhesion to gut tissues and colonization of the alimentary tract of the host, an important stage in the pathogenesis of *Salmonella* (Grzymajlo et al., 2013). A previous study (Yue et al., 2015) suggested a possible correlation between the allelic variation of the amino acid sequence of the FimH protein and bacterial host specificity. FimH protein is involved in regulation of length and mediation of adhesion of type 1 fimbriae and consists of a peptide signal of 22 residues followed by a lectin domain (residues 23–196) and a pilin domain (residues 199–335) connected by a 3-residue link (Yue et al., 2015). In the whole dataset, 19 different missense substitutions have been detected in the lectin domain and 8 different missense substitutions in the pilin domain compared to *S*. Typhimurium’s SL1344 sequence. Although most of the substitutions were reported previously by Yue et al. (2015), 5 new substitutions (residues 91, 112, 113, 252, and 278) were identified in this study. The substitution of the residue 91 in the lectin domain was notably specific to the lineages ST40 and ST39. This new substitution was the only variation that differentiates the FimH protein sequence of the *S*. Derby lineages ST40 and ST39 from that of the *S*. Typhimurium SL1344. The impact of this mutation on host specificity could be investigated by invasion test on epithelial cells. The lineage ST682 shared four identical substitutions (Q89R, L126R, Y131S, and I317N) with *S.* Typhisuis and *S.* Choleraesuis, two serotypes well known for their pathogenesis in pig (Barrow and Methner, 2013). *S.* Typhisuis and *S*. Derby FimH protein sequences differed by only one missense mutation in the peptide signal (K2L) located before the lectin domain (**Figure 5**). FimH adhesin carrying these three substitutions have been shown to present a higher specificity for porcine enterocyte IPEC-J2 than those of *S*. Typhimurium (Yue et al., 2015). The mutations in the ST71 FimH alleles did not relate to any host-specific profiles that have been described in previous study (Yue et al., 2015).

The differences observed between the *fimH* alleles within the different *S*. Derby lineages and other significant SPI substitutions described above, could be used for developing specific real time PCR probe assays aimed at identifying and following the spread of these four *S.* Derby lineages throughout the pork and poultry sectors, from farm to fork.

## Conclusion

The results of this study show that *Salmonella* Derby serotype in France is polyphyletic and can be divided into four distinct lineages distinguished by 14,866 SNPs on average (SD of 12,961). Our results indicate that it should be possible to develop specific and sensitive molecular markers for each lineage. The lineages correspond to the four MLST profiles that have been independently described for *S.* Derby. The dominant lineage in France corresponds to ST40, which is associated with the pork sector. Two other lineages associated with the pork sector are ST39 and ST682. The strains belonging to ST71 are associated with the poultry sector and were isolated from *Gallus gallus*, turkey, but also from duck and guinea fowl food products. These four lineages differ principally by sequence differences in part of the SPI-3, by different allelic composition of the SPI-2 and -5, and by the presence of the pork invasion-related SPI-23 that characterizes the lineages ST40 and ST39. The *S.* Derby strains belonging to ST40 also harbor several AMR genes that are absent from the other STs. Clade 2 of this lineage is characterized by the AMR pattern STR-SSS-TET and likely corresponds to the major PFGE profile described previously by Kerouanton et al. (2013) (profil SDX01). The STR-SSS-TET profile is associated with the presence of an SGI-1 containing a class 1 integron sequence and a mercury resistance gene cluster. Mutations in the lectin and pilin domain of the FimH adhesion protein clearly characterize the different *S*. Derby lineages, and the ST682 presents the same protein lectin and pilin domain sequences than *S.* Typhisuis, causing acute infections in pig.

In this study, the whole-genome-sequencing approach was carried out to provide a high-resolution molecular typing of *S*. Derby strains isolated from the pork and poultry sectors, which can be used to further investigate the host specificity of the four lineages identified. This study constitutes the baseline for identifying over time which genetic lineages were and are present in the livestock and farm environment in France, and will contribute to our understanding of how these lineages can be transmitted to the food industries. Considering source attribution, our data constitute a strong basis for determining which *S*. Derby lineages are responsible for human contamination in France. In that last perspective, we are currently analyzing the genomes of a panel of *Salmonella* Derby isolated from human clinical cases during 2014 and 2015.

## Availability of Data

Genomics sequence assemblies used in this project are available online on the NCBI network under the accession: PRJNA391404 (available at: https://www.ncbi.nlm.nih.gov/bioproject/PRJNA391404). Details for each genomic assembly are summarized in **Supplementary Table S2**, including the accession codes for each genome.

## Author Contributions

SC-S piloted and administered the project. SC-S and YS designed and developed the experiments. YS, SG, and M-LV carried out the experiments and the analyses. SC-S, M-YM, RL, and CF provided acquisitions. YS, SC-S, SG, and M-YM drafted the manuscript. SLH and CF participated in the discussion and reviewed the report.

## Conflict of Interest Statement

The authors declare that the research was conducted in the absence of any commercial or financial relationships that could be construed as a potential conflict of interest.

## Footnotes

1 www.icm-institute.org

2 https://cge.cbs.dtu.dk/services/MLST/

3 https://itol.embl.de/

4 https://blast.ncbi.nlm.nih.gov/Blast.cgi

## Supplementary Material

FIGURE S1 Geographical selection plan for the strains isolated from the pork and poultry sectors.

FIGURE S2 Results of the *in silico* PCR for the SPI-23 into *S.* Derby collection. The *docB* gene corresponds to the coding sequence located immediately after the SPI-23. Only 2014LSAL05133 present small fragments of the SPI-23 (not corresponding to the primers highlighted in this figure) in the ST71.

TABLE S1 Strains information. Sheet 1: Epidata, sample ID, NCBI accession number, genome deep coverage, and breadth. Sheet 2: repartition of the selected strains in each French region. Sheets 3 and 4: selection plan for the strains isolated from the pork and poultry sectors respectively.

TABLE S2 List of the genomes from different serotypes of *Salmonella enterica* subsp. *enterica* used for the FimH sequence analysis. Epidata and sample ID.

TABLE S3 List of strains presenting antimicrobial resistance genes.
