# Supplementary figures and images for: Polyphyletic Nature of Salmonella enterica Serotype Derby and Lineage-Specific Host-Association Revealed by Genome-Wide Analysis
Source: Front Microbiol. 2018 May 17;9:891. doi: 10.3389/fmicb.2018.00891 (PMC5966662; doi:10.3389/fmicb.2018.00891)

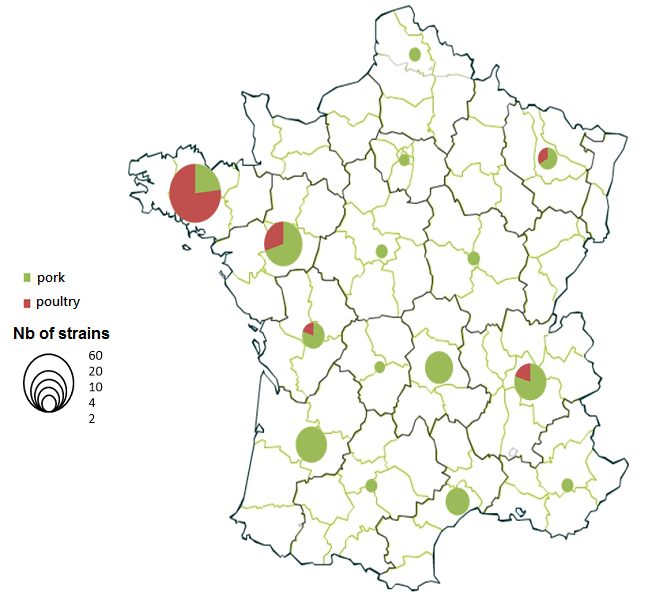

Supplement: FIGURE S1 — Geographical selection plan for the strains isolated from the pork and poultry sectors. [file Image_1.TIFF]

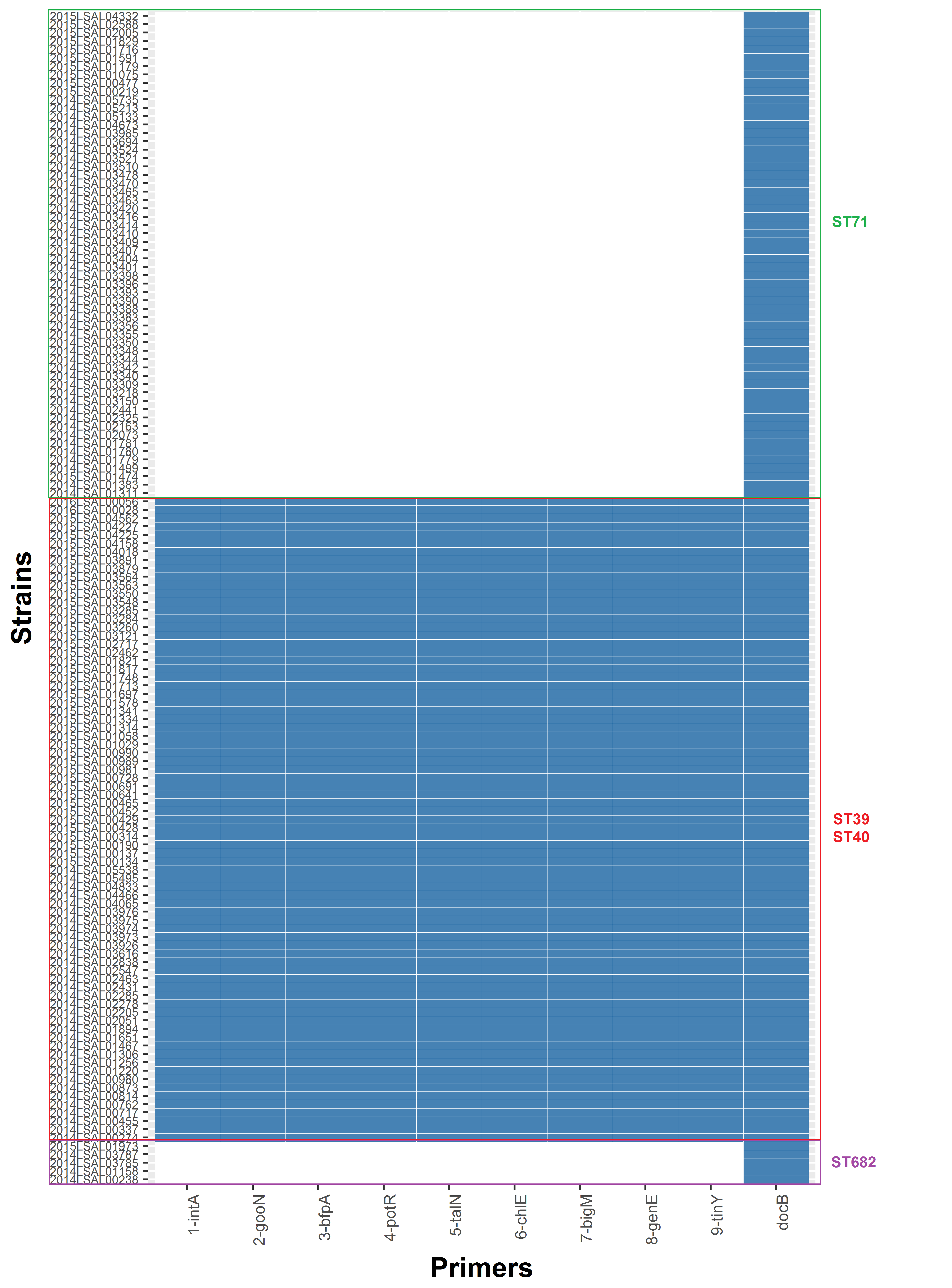

Supplement: FIGURE S2 — Results of the in silico PCR for the SPI-23 into S. Derby collection. The docB gene corresponds to the coding sequence located immediately after the SPI-23. Only 2014LSAL05133 present small fragments of the SPI-23 (not corresponding to the primers highlighted in this figure) in the ST71. [file Image_2.TIF]
